# Latent trajectories of early social communication development are associated with autism diagnosis and language outcomes

**DOI:** 10.3389/frcha.2026.1723743

**Published:** 2026-04-09

**Authors:** Laura A. Edwards, Scott Gillespie, Laura M. Johnson, Moira L. Pileggi, Warren Jones, Ami Klin

**Affiliations:** 1 Department of Pediatrics, Emory University School of Medicine, Atlanta, GA, United States; 2 Marcus Autism Center, Children’s Healthcare of Atlanta, Atlanta, GA, United States

**Keywords:** autism, developmental disability, language, latent classes, social communication, longitudinal trajectory

## Abstract

Social communication, or a child’s ability to interact with others using verbal and nonverbal cues, is a strong predictor of later language development and lifelong outcomes in individuals on the autism spectrum. Interventions that target social communication skills would benefit from careful analysis of the emergence of these skills from their developmental onset. The current study assessed social communication in 801 infants and toddlers using the Communication and Symbolic Behavior Scales (CSBS) at 12, 15, 18, and 24 months of age. Standard social, speech, and symbolic composite scores were modelled in parallel using latent class analysis and growth mixture modeling, to identify distinct classes of children based on social communication trajectories. Class membership was then associated with sex and diagnostic outcome, and associations between 24-month language and social communication trajectory class membership were examined. Secondarily, associations between school age social disability outcomes and early trajectory class membership were examined in a subsample for whom follow-up measures were collected. Two classes of social and symbolic composite trajectories, and 3 classes of speech trajectories were identified. Classes were strongly associated with sex and diagnosis, as well as 24-month language outcomes, controlling for diagnosis and maternal education. Associations between class membership and school age social disability were not robust to controls for diagnosis and maternal education. Across all domains, differences present at 12 months of age persisted or increased through 24 months. Our findings indicate specific trajectories of social communication development which may benefit from interventions targeting social communication skills from around the first birthday. Such interventions would also promote positive development of both receptive and expressive language abilities. Our findings also demonstrate complexity and heterogeneity in development across the breadth of social communication skills, as well as within diagnostic categories. Early assessment of social communication skills—and interventions or educational services aimed at supporting positive development in potentially vulnerable domains—may thus prove beneficial for children before diagnosis, and/or regardless of familial or other likelihood for autism or other developmental delays and disorders.

## Introduction

Social communication refers to a child's ability to interact with others using verbal and nonverbal cues. In typical development, foundational social communication behaviors can be observed from birth, when newborns preferentially orient to speech over synthetic sounds, caregivers over strangers, faces over non-faces, and direct over averted gaze (1–5). Social communication abilities continue to develop before the acquisition of spoken language, with social smiling being observed between 6 and 8 weeks of age, reciprocal social babbling emerging around 6 months, and giving and showing behaviors emerging in concert with growing motor control around 9 months. Between 9 and 12 months of age, infants begin to interact with others using facial expressions, eye contact, gestures, and vocalizations (6, 7). These prelinguistic skills set the stage for the acquisition of first words around 12 months of age, and the subsequent acceleration of spoken language development that continues through the second year of life (8, 9).

Departures from these typical social communication milestones may indicate or contribute to the development of communication disorders or developmental disabilities such as autism spectrum disorder (ASD). ASD is a neurodevelopmental condition characterized by challenges in social communication, and restricted, repetitive patterns of behavior (10). Toddlers with ASD have a limited repertoire of prelinguistic skills (11–13), exhibit lower rates of communication and fewer gestures (11, 14), as well as fewer instances of joint attention, social referencing, and shared positive affect (15–18), than their typically developing peers; some of these differences are detectable from the first year of life (19–22). Early observable behavioral differences in social communication often consolidate into a diagnosable behavioral phenotype of autism around 24- to 36-months-of-age (23, 24).

Social communication development is a strong predictor of later language development. In ASD, language abilities are highly heterogeneous, and delays are pervasive. While many children on the autism spectrum enter preschool with language delays and slowly acquire language during the preschool years, developing some spoken words or phrase speech by kindergarten (25–27), about 30% of children on the autism spectrum fail to acquire any spoken language (28). Language and communication difficulties are also increased among children with increased likelihood for autism (IL) due to family history, even in the absence of developing autism (29, 30). Language, in turn, is a strong and consistent predictor of lifelong outcomes in individuals on the autism spectrum. More specifically, receptive language in preschool is associated with long term social functioning and adaptive skills in autism (31, 32); and better expressive language skills upon kindergarten entry predict higher social and vocational success, including literacy development, school achievement, and independence in adulthood, in individuals with ASD (32–38). The autism community has identified fostering better language skills as a priority for interventions (39, 40).

Past research has parsed and investigated heterogeneity in early language development, primarily for the purpose of earlier ASD diagnosis. In a study using latent class growth analysis to examine the development of siblings of children with autism between 6 and 36 months, Landa et al. (52) identified four classes of motor, language, and nonverbal development—an accelerated class, a normative class, a language and motor delayed class, and a class showing widespread delays in skill acquisition; children with autism diagnoses were distributed across the latter three classes. Riva et al. (53) also investigated siblings of children with autism using similar methods and identified four classes of expressive language trajectories between 12 and 24 months; these trajectories in combination with gesture development were predictive of 2 year old ASD symptoms. In a large study of children with and without siblings with autism, Longard et al. (54) used semi-parametric group based modelling to examine expressive and receptive language trajectories from the first to the third year of life, and identified three distinct trajectories (within both expressive and receptive language development); the trajectories representing declining language abilities in particular were associated with an ASD diagnosis at 36 months.

Additional research has parsed and investigated heterogeneity in language abilities and development in individuals with ASD and related disabilities with the aim of improving language outcomes by identifying efficacious targets and timing for language interventions. Broome et al. (55) used hierarchical cluster analysis to create subgroups of autistic children based on speech profiles, and investigated the stability of these subgroups over 12 months; they found that some children who presented with limited language and speech capacity at baseline improved across all communication variables and were talking after 12 months, whereas others remained nonverbal; consonant inventory at baseline was associated with speech outcomes. Frazier et al. (56) used growth mixture modeling to examine longitudinal language trajectories in children with ASD undergoing early intensive behavioral intervention, and identified three latent classes, or sub-groups, of children, based on their language scores over the course of the intervention—one with low baseline scores and little improvement over time, one with low baseline scores that showed substantial increases over time, and one with average initial scores, but varying trajectories depending on the exact language measure under study; they further showed that earlier age at intervention start, higher baseline cognitive ability, and lower ASD symptomatology were associated with better language trajectories. In a study of siblings of children with and without ASD, growth curve analysis revealed that 4-year-old language (but not cognitive) scores were lower in siblings of ASD compared to siblings of children without ASD, and 40% of siblings of ASD (compared to 16% of siblings of children without ASD) showed language, cognitive, or academic difficulties at 7 years old (57).

The aforementioned studies identify persistent heterogeneity in language abilities in children with ASD and related disabilities, as well as meaningful predictors thereof, and thus demonstrate need and potential targets for language interventions. However, research directly parsing the heterogeneity in social communication skills and language outcomes in children with ASD as well as related communication disabilities, or investigating developmental trajectories of social communication skills preceding ASD diagnosis, is more limited. Yet social communication skills are ideal targets for early intervention, because of the early emergence of these challenges in autism, and their impact on language development. Interventions aimed at improving language in ASD would benefit from careful analysis of the emergence of social-communication skills from their developmental onset, as well as close attention to the specific social communication domains or skills that are most strongly predictive of later positive social and language development. To date, studies reporting on early social communication vulnerabilities and their relationship to later language have tended to focus on a limited number of specific social communication behaviors, such as gestures (41), or joint attention (42), and past work has not examined the specificity of ASD social communication trajectories relative to those of children who are diagnosed with non-autism developmental delays or disorders.

The current study aims to address these gaps in the literature, by identifying distinct, latent trajectory classes of early social communication development between 12 and 24 months of age, and evaluating associations between these social communication trajectory classes, and later language and social outcomes. Social communication skills are profiled using a multidomain, standardized, clinical behavioral assessment. Since social communication and language are heterogeneous across and even within individuals with and without autism over time, we employ latent class analysis [LCA (43)] and growth mixture modeling approaches to identify early, developmental, transdiagnostic vulnerabilities in social communication that affect later language and social development. The findings herein may inform more precise design, timing, and targets of early interventions (or pre-emptive interventions) aimed at optimizing lifelong outcomes in children with autism and/or associated developmental disabilities.

## Materials and methods

### Participants and study design

Infants and toddlers were recruited to participate in studies mapping social development from birth through early toddlerhood at Emory University and the Marcus Autism Center in Atlanta, GA, and the Yale Child Study Center, New Haven, CT, USA. To maximize our sample sizes, the analyses herein include data from several studies over a wide range of time (between 2007 and 2020), during which infants and toddlers were assessed, and often followed longitudinally, between birth and 36 months of age. Many of these studies classified children as increased familial likelihood (IL) for autism if they had an immediate family member on the autism spectrum and infants were designated low likelihood (LL) if they had no autistic family members within three degrees (44, 45). Infants and toddlers were excluded from participation if they met any of the following exclusionary criteria: gestational age below 34 weeks, neonatal hearing impairment, or non-febrile seizure disorders. Additionally, our consent process required proficiency in English, so caregivers of the participants in our sample were primarily English-speaking. Research protocols were approved by the Emory and Yale University Institutional Review Boards, and all caregivers provided written informed consent for their infant's participation upon enrollment.

The following measures were collected at the timepoints indicated:

#### Sociodemographic characteristics

At study intake, parents of participating infants and toddlers filled out demographic questionnaires, including information about their child's sex, race, ethnicity, family, educational, and health history. Varied indicators of family socioeconomic status (SES) were collected across studies; maternal education is reported and included in applicable analyses herein as it was the SES indicator most frequently collected across included participants, and is the component of SES most strongly related to child development outcomes (46, 47).

#### Communication and Symbolic Behavior Scales (CSBS)

Social communication was assessed at children's 12-, 15-, 18-, and 24-month-old visits using the Communication and Symbolic Behavior Scales (CSBS) behavior sample (48)—a multidomain, standardized, communication assessment. The CSBS is a semistructured behavior sample comprised of communicative temptations and play probes with a trained examiner to assess communication skills and indicators of symbolic development, including vocalizations, gestures, and facial expressions, across a series of interaction probes. CSBS social, speech, and symbolic composite standard scores were modelled in parallel using LCA, to identify distinct classes of children based on social communication trajectories. The CSBS social composite aggregates indicators of emotion and eye gaze, communication, and gestures; the speech composite aggregates indicators of sounds and words produced, and the symbolic composite aggregates indicators of understanding and object use.

#### Mullen Scales of Early Learning

Expressive Language (EL) and Receptive Language (RL) age equivalent (AE) scores were obtained from the MSEL (58), which was administered by a trained clinician at the 24-month visit. The MSEL, which is normed for children between 1 and 68 postnatal months, is used extensively in research to assess language development and demonstrates good construct, convergent, and divergent validity across a variety of developmental presentations (59). AE scores were utilized in the present analyses, as has been done previously [e.g. (25, 60–62)].

#### Diagnostic classification

Each child's diagnostic classification was determined by clinical best estimate (CBE) of expert clinicians, including licensed psychologists and speech-language pathologists on the clinical assessment teams at Marcus Autism Center and the Yale Child Study Center. Comprehensive CBE, which considers results of all characterization procedures and developmental history, is the most reliable diagnostic procedure in this age range. The current analyses include children with CBE diagnostic classifications of autism spectrum disorder (ASD), typical development (TD), and non-autism developmental delays and disorders (non-AUT). Non-autism developmental delays and disorders included a range of cognitive and communication delays, psychiatric disorders, and delays due to known genetic disorders. For the analyses herein, this heterogeneity was parsed into the following subgroups: non-autism developmental delays (such as cognitive, language, or communication delays; DD); subthreshold non-autism developmental delays (subthreshold DD); subthreshold autism symptoms or the broader autism phenotype [Subthreshold ASD/BAP (29, 49)]; Psychiatric Conditions (e.g., anxiety, selective mutism); and Known Genetic Disorders (such as MECP-2 duplication, Williams syndrome)^1^.

#### Social Responsiveness Scale, 2nd edition (SRS-2)

Participants who completed the measures above at the Marcus Autism Center were also invited to participate in an (ongoing) follow-up study of their school age outcomes. As part of this study, participants' caregivers completed the Social Responsiveness Scale, 2nd Edition (SRS-2). The SRS-2 is a 65-item questionnaire that is used to identify and quantify social impairment associated with autism. The School-Age form (designed for 4–18 year olds) was used in this study. It takes between 15 and 20 min to complete, and produces a total score, as well as subscale scores for awareness, cognition, communication, motivation, and restricted interests and repetitive behavior. SRS-2 Total T scores (*M* = 50, *SD* = 10) are used to aid clinical interpretation and were used in the current analyses.

Children were included in the analyses herein if they had at least one CSBS assessment between 12 and 24 months. A total of 801 children (*N* = 286 females; *N* = 515 males) were included in our sample.

### Statistical analyses

Sociodemographic characteristics were summarized using means and standard deviations for continuous variables and frequencies and percentages for categorical variables.

Using each of the three CSBS composite standard scores—social, speech, and symbolic—measured at 12, 15, 18, and 24 months^2^ we applied growth mixture modeling (GMM) to identify distinct developmental trajectories in early social communication. GMM is a longitudinal modeling approach that identifies unobserved subgroups of individuals (latent classes) who follow similar growth patterns over time. Unlike latent class growth analysis (LCGA), which assumes no variation in trajectories within classes, GMM allows for individual-level variability within each class by incorporating random effects. Growth mixture modeling was conducted using FIML to include participants with incomplete longitudinal data. Over half of participants contributed data at only one timepoint, which were distributed across 12, 15, 18, and 24 months. In our models, random intercepts were included, but random slopes were not retained due to lack of significance. A quadratic function of time was used to capture potential non-linear growth.

We used an iterative model-building strategy, beginning with a one-class model and increasing the number of classes stepwise. Model fit was evaluated using several criteria, including the Akaike Information Criterion (AIC), adjusted Bayesian Information Criterion (adjusted BIC), and entropy. Lower AIC and adjusted BIC values indicate better fit. Entropy values range from 0 to 1, with higher values reflecting greater certainty in class assignment and values >0.80 indicate good classification (63). To determine the optimal number of classes, the primary stopping rule was the LMR-LRT: we retained the largest number of classes for which the LMR-LRT was statistically significant, provided that the resulting solution was clinically interpretable and that class sizes were sufficient. When indices disagreed, priority was given to the LMR-LRT in combination with theoretical interpretability and class size, rather than solely to AIC, BIC, or entropy (70).

After assigning individuals to latent classes, we evaluated associations between class membership and child sociodemographic characteristics and diagnostic classification. Group differences in continuous variables were tested using *t*-tests or ANOVA, while chi-square tests were used for categorical variables. Child sociodemographic characteristics included sex, race/ethnicity, maternal education^3^, and familial likelihood for autism; diagnostic classification refers to CBE-based diagnoses at 24 and/or 36 months of age. We then evaluated associations between class membership and language using *t*-tests or ANOVA, as well as multivariable regression analyses adjusting for diagnostic classification and maternal education [given established links between socioeconomic status and language development (50, 51)]. Language outcomes included expressive (EL) and receptive language (RL) at 24 months, measured by Mullen Scales of Early Learning (MSEL) age-equivalent scores. In children for whom followup social disability outcome measures were taken at school-age, we also evaluated associations between these scores and social communication latent class membership using *t*-tests or ANOVA, as well as multivariable regression analyses adjusting for diagnostic classification. Social outcomes were operationalized as school-age (5–7 years) social disability, measured using the Social Responsiveness Scale-2 (SRS-2) Total T score.

All analyses were conducted using SAS v9.4 (SAS Institute, Cary, NC) and Mplus v8 (Muthén & Muthén, Los Angeles, CA).

## Results

### Participant characteristics

Table 1 displays the breakdown of sociodemographic and clinical characteristics across the entire sample. Sociodemographic and clinical characteristics specific to each site (Yale and Emory) are broken down in Supplementary Table S1. Race distributions varied across site, likely due to the distinct demographics of the study locations; further site-based details are discussed in Supplementary Materials. Data from both sites were combined in the current manuscript in order to capitalize on heterogeneity and increase robustness of the analyses.

**Table 1:** Demographics and clinical information (*N* = 801).

Characteristics	N (%) or Mean (SD)
Child Sex	
Female	286 (35.7%)
Male	515 (64.3%)
Child Race	
White	585 (73%)
Black	100 (12.5%)
Multi-racial or Other	76 (9.5%)
Asian	23 (2.9%)
American Indian or Alaskan Native	2 (0.2%)
Unknown or Not Reported	15 (1.9%)
Child Ethnicity	
Hispanic	76 (9.5%)
Not Hispanic	706 (88.1%)
Unknown or Not Reported	19 (2.4%)
Maternal Education	
HS/GED or Less	75 (9.4%)
Associate's degree/Some College	182 (22.7%)
Bachelor's degree	135 (16.8%)
Master's degree	139 (17.3%)
PhD/Professional degree	68 (8.5%)
Unknown or Not Reported	202 (25.2%)
Age at Mullen (Months), *N* = 366	24.44 (1.08)
Age at SRS (Months), *N* = 117	83.94 (9.86)
Diagnosis	
ASD	241 (30.1%)
TD	335 (41.8%)
DD	104 (13%)
Subthreshold DD	8 (1%)
Subthreshold ASD/BAP	74 (9.2%)
Psychiatric Conditions	11 (1.4%)
Known Genetic Disorders	15 (1.9%)
Unknown or Not Reported	13 (1.6%)
Familial Likelihood	
IL	241 (30.1%)
LL	366 (45.7%)
Unknown or Not Reported	194 (24.2%)
CSBS Data Completeness (# of Timepoints)	
One (1) timepoint	417 (52.1%)
Two (2) timepoints	189 (23.6%)
Three (3) timepoints	167 (20.9%)
Four (4) timepoints	28 (3.5%)

### Latent class solutions from quadratic growth mixture models

#### Social composite

**Figure 1:**
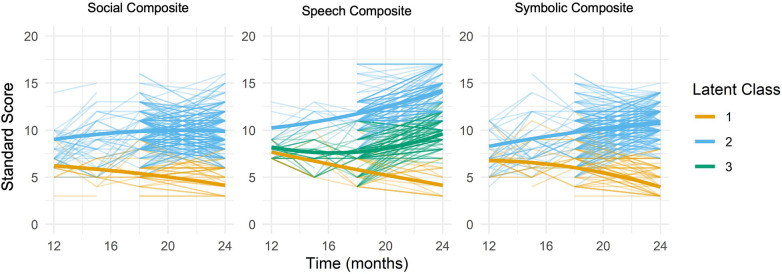
Spaghetti plots of CSBS standard score composites by latent class with quadratic regression overlay.

Based on fit indices (Table 2), the optimal solution for CSBS social composite trajectories was 2 classes; although the AIC and adjusted-BIC decreased when comparing the three-class solution to the two-class solution, the statistical values of LMR-LRT and BLRT were not significant. Figure 1 illustrates the two trajectories for CSBS social composite standard scores: a higher increasing trajectory (67.5%; Class 2), and a lower decreasing trajectory (32.5%; Class 1). Table 3 shows that mean social composite standard scores significantly differed between Classes 1 and 2 at all time points. With regard to sociodemographic characteristics, social composite trajectory class membership was significantly associated with (1) sex (*p* < 0.001), such that Class 2 contained a larger proportion of females and lower proportion of males than Class 1; (2) race (*p* < 0.001) and ethnicity (*p* = 0.016) such that Class 1 contained lower proportions of White and higher proportions of Black, Multi-racial or Other, and Hispanic participants than Class 2; and (3) maternal education (*p* < 0.001) such that Class 2 contained higher proportions of participants whose mothers had completed college compared to Class 1. Social trajectory class membership was also significantly associated with familial likelihood and diagnosis (both *p* < 0.001): Class 2 contained larger proportions of LL compared to IL children, whereas Class 1 showed the reverse pattern; Class 2 was primarily (60.8%) TD at outcome, whereas Class 1 was comprised of majority (74.2%) ASD at outcome. Class 2 also contained higher proportions of all non-AUT developmental delays and disorders groups, except for Known Genetic Disorders, than Class 1.

**Table 2:** LCA model fit statistics for CSBS subscale scores (*N* = 801).

LCA Solution	# Classes	Log-Likelihood	AIC	Adjusted-BIC	Class Quality	k-1 latent classes tests		Latent Class Sizes
						Adjusted LMR Test	Bootstrapped LRT	
Social SS	1	-3,388.8	6,787.6	6,795.1	NA	NA	NA	801
	2*	−3,347.9	6,713.8	6,727.4	0.64	**<0.001**	**<0.001**	260; 541
	3	−3,332.3	6,690.6	6,710.2	0.66	0.141	0.429	503; 29; 269
								
Speech SS	1	−3,508.6	7,027.1	7,034.7	NA	NA	NA	801
	2	−3,417.9	6,853.7	6,867.3	0.55	**<0.001**	**<0.001**	261; 540
	3*	−3,370.8	6,767.7	6,787.3	0.63	**<0.001**	**<0.001**	163; 249; 389
	4	−3,347.2	6,728.4	6,754.0	0.64	**<0.001**	**<0.001**	157; 139; 306; 199
	5	−3,330.7	6,703.3	6,735.0	0.68	0.260	0.265	203; 117; 159; 16; 306
								
Symbolic SS	1	−3,474.9	6,959.8	6,967.3	NA	NA	NA	801
	2*	−3,335.9	6,689.8	6,703.4	0.70	**<0.001**	**<0.001**	313; 488
	3	−3,335.9	6,697.8	6,717.4	0.43	0.943	1.000	417; 27; 357

^a^ Models estimated using growth mixture modeling (GMM) with latent class growth analysis (LCGA) and a random intercept, incorporating quadratic time effects for each outcome.

^b^ Adjusted Lo-Mendell-Rubin likelihood ratio test (LMR) and Bootstrapped likelihood ratio test (BLRT) compare the *k*-class model to the *(k – 1)*-class model.

^c^ Classification quality measured by entropy (range: 0–1, higher values indicate better separation of classes).

^d^ Latent class sizes reported as number of participants per class; an asterisk (*) indicates the model selected as the best-fitting solution for that outcome.

^e^ Findings significant at alpha = 0.05 are bolded.

**Table 3:** Latent class analysis of social SS.

Characteristic N (%) or Mean (SD)	N	Class 1, *N* = 260	Class 2, *N* = 541	*P*-Value
LCA Features				
Time 12	327	5.80 (1.50)	9.03 (2.08)	**<0.001**
Time 15	106	5.17 (1.26)	9.42 (2.49)	**<0.001**
Time 18	411	5.03 (1.58)	9.75 (1.96)	**<0.001**
Time 24	564	4.15 (1.34)	9.77 (2.14)	**<0.001**
Class 1 Probability	801	88% (14%)	9% (13%)	**<0.001**
Class 2 Probability	801	12% (14%)	91% (13%)	**<0.001**
Number observations	801	1.32 (0.73)	1.97 (0.9)	**<0.001**
Demographics and Clinical Features				
Sex	801			**<0.001**
Female		67 (25.8%)	219 (40.5%)	
Male		193 (74.2%)	322 (59.5%)	
Child Race	801			**<0.001**
White		154 (59.2%)	431 (79.7%)	
Black		53 (20.4%)	47 (8.7%)	
Multi-racial or Other		38 (14.6%)	38 (7%)	
Asian		7 (2.7%)	16 (3%)	
American Indian or Alaskan Native		1 (0.4%)	1 (0.2%)	
Unknown or Not Reported		7 (2.7%)	8 (1.5%)	
Child Ethnicity	801			**0.016**
Hispanic		30 (11.5%)	46 (8.5%)	
Not Hispanic		219 (84.2%)	487 (90%)	
Unknown or Not Reported		11 (4.2%)	8 (1.5%)	
Maternal Education	599			**<0.001**
Less than college graduate		121 (51.5%)	136 (37.4%)	
College graduate		114 (48.5%)	228 (62.6%)	
Diagnosis	801			**<0.001**
ASD		193 (74.2%)	48 (8.9%)	
TD		6 (2.3%)	329 (60.8%)	
DD		24 (9.2%)	80 (14.8%)	
Subthreshold DD		0 (0%)	8 (1.5%)	
Subthreshold ASD/BAP		18 (6.9%)	56 (10.3%)	
Psychiatric Conditions		2 (0.8%)	9 (1.7%)	
Known Genetic Disorders		12 (4.6%)	3 (0.5%)	
Unknown or Not Reported		5 (1.9%)	8 (1.5%)	
Familial Likelihood	801			**<0.001**
IL		58 (22.3%)	183 (33.8%)	
LL		43 (16.5%)	323 (59.7%)	
Unknown or Not Reported		159 (61.2%)	35 (6.5%)	

^a^ *p*-values are based on two-sample t-tests (continuous) or Monte Carlo–estimated Fisher's exact tests (categorical).

#### Speech composite

Table 2 shows that the 3-class solution had lower AIC and adjusted BIC, a statistically significant improvement in model fit (on both the LMR and BLRT significance tests), and higher entropy compared to the 2-class solution. We also explored 4-class and 5-class solutions, and while the 4-class version met the LMR-LRT significance criterion laid out above, visual inspection of this solution (Supplementary Figure S1), revealed two similar, increasing trajectories. Additional analyses (Supplementary Table S2) indicated that these two classes were not significantly differentiated by demographic characteristics, suggesting that the fourth class did not provide substantively new clinical information. Therefore, considering the quantitative metrics along with the clinical relevance of the model, we selected the 3-class solution as the best-fitting model for CSBS speech composite standard score trajectories. Figure 1 illustrates these three classes: a higher increasing trajectory (31.1%; Class 2), a lower decreasing trajectory (20.3%; Class 1), and a medium magnitude parabolic trajectory (48.6%; Class 3). Table 4 shows that mean speech composite standard scores significantly differed between classes at all time points, starting at 12 months old. With regard to sociodemographic characteristics, speech composite trajectory class membership was also significantly associated with sex, race, and maternal education (all *p*s < 0.001), such that Class 2 contained a larger proportion of females, White participants, and college graduate mothers, and lower proportions of males, Black and Multi-racial or Other participants, and mothers without college degrees than Classes 3 and 1, respectively. Speech trajectory class membership was also significantly associated with familial likelihood and diagnosis (*p*s < 0.001): Class 2 contained larger proportions of TD and lower proportions of ASD children than Classes 3 and 1 respectively. Of the 3 speech composite classes, Class 3 contained the highest proportion of children in the DD, subthreshold ASD/BAP and Psychiatric Conditions groups, while Class 1 had the highest proportion of children with Known Genetic Disorders. *post-hoc* pairwise comparisons between these 3 speech classes, presented in Supplementary Table S3, show that the aforementioned sociodemographic and clinical characteristics differ significantly across all combinations of the 3 classes, with the exception of sex, which is not distributed significantly differently across Classes 2 vs. 3; and ethnicity, which only differs between Classes 1 and 2.

**Table 4:** Latent class analysis of speech SS.

Characteristic N (%) or Mean (SD)	N	Class 1, *N* = 163	Class 2, *N* = 249	Class 3, *N* = 389	*P*-Value
LCA Features					
Time 12	327	7.38 (0.86)	10.28 (2.13)	8.11 (1.16)	**<0.001**
Time 15	106	6.50 (1.98)	11.38 (1.61)	7.66 (1.57)	**<0.001**
Time 18	411	4.89 (1.39)	11.65 (1.77)	7.67 (1.73)	**<0.001**
Time 24	564	3.93 (1.20)	14.19 (1.85)	9.56 (1.72)	**<0.001**
Class 1 Probability	801	88% (15%)	1% (4%)	13% (15%)	**<0.001**
Class 2 Probability	801	0% (0%)	86% (17%)	10% (13%)	**<0.001**
Class 3 Probability	801	12% (15%)	13% (15%)	77% (17%)	**<0.001**
Number observations	801	1.29 (0.72)	1.93 (0.78)	1.85 (0.97)	**<0.001**
Demographics and Clinical Features					
Sex	801				**<0.001**
Female		38 (23.3%)	107 (43%)	141 (36.2%)	
Male		125 (76.7%)	142 (57%)	248 (63.8%)	
Child Race	801				**<0.001**
White		90 (55.2%)	220 (88.3%)	275 (70.7%)	
Black		34 (20.9%)	12 (4.8%)	54 (13.9%)	
Multi-racial or Other		27 (16.6%)	8 (3.2%)	41 (10.5%)	
Asian		6 (3.7%)	6 (2.4%)	11 (2.8%)	
American Indian or Alaskan Native		1 (0.6%)	0 (0%)	1 (0.3%)	
Unknown or Not Reported		5 (3.1%)	3 (1.2%)	7 (1.8%)	
Child Ethnicity	801				**0.219**
Hispanic		17 (10.4%)	22 (8.8%)	37 (9.5%)	
Not Hispanic		139 (85.3%)	225 (90.4%)	342 (87.9%)	
Unknown or Not Reported		7 (4.3%)	2 (0.8%)	10 (2.6%)	
Maternal Education	599				**<0.001**
Less than college graduate		84 (54.9%)	34 (29.3%)	139 (42.1%)	
College graduate		69 (45.1%)	82 (70.7%)	191 (57.9%)	
Diagnosis	801				**<0.001**
ASD		130 (79.7%)	8 (3.2%)	103 (26.5%)	
TD		5 (3.1%)	188 (75.5%)	142 (36.5%)	
DD		14 (8.6%)	15 (6%)	75 (19.3%)	
Subthreshold DD		0 (0%)	5 (2%)	3 (0.8%)	
Subthreshold ASD/BAP		6 (3.7%)	24 (9.6%)	44 (11.3%)	
Psychiatric Conditions		0 (0%)	4 (1.6%)	7 (1.8%)	
Known Genetic Disorders		8 (4.9%)	2 (0.8%)	5 (1.3%)	
Unknown or Not Reported		0 (0%)	3 (1.2%)	10 (2.6%)	
Familial Likelihood	801				**<0.001**
IL		19 (11.7%)	102 (41%)	120 (30.8%)	
LL		27 (16.6%)	137 (55%)	202 (51.9%)	
Unknown or Not Reported		117 (71.8%)	10 (4%)	67 (17.2%)	

^a^ *p*-values are based on two-sample t-tests (continuous) or Monte Carlo–estimated Fisher's exact tests (categorical).

#### Symbolic composite

Table 2 shows that the 2-class solution for symbolic standard score trajectories exhibited best model fit and entropy. Based on these criteria, along with the clinical relevance of the model, we selected the 2-class solution for CSBS symbolic composite standard score trajectories. Figure 1 shows these two classes: a higher increasing trajectory (60.9%; Class 2), and a lower decreasing trajectory (39.1%; Class 1). Table 5 shows that mean symbolic composite standard scores significantly diverged between these two classes at 12, 15, 18, and 24 months. Symbolic composite trajectory class membership was significantly associated with sex, race and ethnicity and maternal education (all *p*s < 0.001), such that Class 2 contained a larger proportion of females, White and Non-Hispanic participants, and mothers with college degrees compared to Class 1. Symbolic trajectory class membership was also significantly associated with familial likelihood and diagnosis (both *p*s < 0.001): Class 2 contained larger proportions of LL and TD and lower proportions of IL and ASD children, compared to Class 1. The distribution of children with non-AUT developmental delays and disorders varied across these two classes, with Class 1 containing higher proportions of children with DD and Known Genetic Disorders, and Class 2 containing higher proportions of children with subthreshold DD, subthreshold ASD/BAP, and Psychiatric Conditions.

**Table 5:** Latent class analysis of symbolic SS.

Characteristic N (%) or Mean (SD)	N	Class 1, *N* = 313	Class 2, *N* = 488	*P*-Value
LCA Features				
Time 12	327	6.59 (2.04)	8.36 (2.04)	**<0.001**
Time 15	106	6.20 (1.84)	9.68 (2.51)	**<0.001**
Time 18	411	6.06 (1.65)	9.83 (2.17)	**<0.001**
Time 24	564	4.13 (1.34)	10.75 (1.73)	**<0.001**
Class 1 Probability	801	90% (15%)	9% (13%)	**<0.001**
Class 2 Probability	801	10% (15%)	91% (13%)	**<0.001**
Number observations	801	1.40 (0.79)	1.99 (0.89)	**<0.001**
Demographics and Clinical Features				
Sex	801			**<0.001**
Female		86 (27.5%)	200 (41%)	
Male		227 (72.5%)	288 (59%)	
Child Race	801			**<0.001**
White		189 (60.4%)	396 (81.1%)	
Black		62 (19.8%)	38 (7.8%)	
Multi-racial or Other		44 (14.1%)	32 (6.6%)	
Asian		9 (2.9%)	14 (2.9%)	
American Indian or Alaskan Native		1 (0.3%)	1 (0.2%)	
Unknown or Not Reported		8 (2.6%)	7 (1.4%)	
Child Ethnicity	801			**<0.001**
Hispanic		41 (13.1%)	35 (7.2%)	
Not Hispanic		259 (82.8%)	447 (91.6%)	
Unknown or Not Reported		13 (4.1%)	6 (1.2%)	
Maternal Education	599			**<0.001**
Less than college graduate		144 (52.0%)	113 (35.1%)	
College graduate		133 (48.0%)	209 (64.9%)	
Diagnosis	801			**<0.001**
ASD		195 (62.3%)	46 (9.4%)	
TD		23 (7.3%)	312 (63.9%)	
DD		52 (16.6%)	52 (10.7%)	
Subthreshold DD		1 (0.3%)	7 (1.4%)	
Subthreshold ASD/BAP		23 (7.3%)	51 (10.5%)	
Psychiatric Conditions		4 (1.3%)	7 (1.4%)	
Known Genetic Disorders		12 (3.8%)	3 (0.6%)	
Unknown or Not Reported		3 (1%)	10 (2.1%)	
Familial Likelihood	801			**<0.001**
IL		77 (24.6%)	164 (33.6%)	
LL		74 (23.6%)	292 (59.8%)	
Unknown or Not Reported		162 (51.8%)	32 (6.6%)	

^a^ *p*-values are based on two-sample t-tests (continuous) or Monte Carlo–estimated Fisher's exact tests (categorical).

Details of the LCA quadratic regression parameters for each composite are available in Supplementary Table S4.

### Associations between social communication latent classes and language outcomes

#### Social composite latent classes

Social composite class membership was associated with both RL and EL AE scores at 24 months, such that, on average, receptive and expressive language were significantly lower, and delayed relative to age-based norms in Class 1, whereas average Class 2 RL and EL 24-month AE scores were higher and slightly elevated relative to age-based norms. These results were robust to controls for maternal education and diagnosis (Table 6).

**Table 6:** Mullen Scales of Early Learning outcomes by latent class solutions, unadjusted and adjusted for demographic and clinical class differences (maternal education and child diagnosis).

Outcome	Unadjusted, LS-Mean (95% CI)				Adjusted, LS-Mean (95% CI)			
	Class 1	Class 2	Class 3	*P*-Value	Class 1	Class 2	Class 3	*P*-Value
Social SS								
Mullen RL	16.5 (14.2, 18.8)	27.0 (26.5, 27.6)	NA	**<0.001**	17.8 (15.3, 20.3)	24.3 (22.5, 26.0)	NA	**<0.001**
Mullen EL	17.9 (15.4, 20.4)	26.4 (25.7, 27.1)	NA	**<0.001**	18.5 (15.6, 21.5)	22.4 (20.2, 24.5)	NA	**0.006**
Speech SS								
Mullen RL	15.7 (12.3, 19.0)	28.2 (27.5, 28.8)	24.7 (23.7, 25.6)	**<0.001**	17.1 (14.2, 20.0)	24.6 (22.8, 26.4)	22.9 (21.2, 24.6)	**<0.001**
Mullen EL	13.1 (10.5, 15.7)	29.1 (28.3, 29.9)	23.0 (22.1, 23.9)	**<0.001**	13.4 (10.5, 16.3)	26.5 (24.4, 28.6)	21.4 (19.5, 23.3)	**<0.001**
Symbolic SS								
Mullen RL	17.9 (16.0, 19.7)	27.7 (27.3, 28.2)	NA	**<0.001**	18.2 (16.2, 20.2)	25.7 (23.9, 27.4)	NA	**<0.001**
Mullen EL	18.1 (16.7, 19.6)	27.1 (26.4, 27.8)	NA	**<0.001**	18.3 (16.1, 20.5)	23.9 (21.8, 26.0)	NA	**<0.001**

^a^ LS-means and 95% CI are based on linear regression models. “Adjusted” models control for maternal education and child diagnosis. Mullen outcomes: *N* = 366. NA = class not present for the outcome measure. CSBS assessment covers years 2007–2020. In a sensitivity analysis restricted to participants with complete covariate data, adjusted associations were consistent in strength, direction, and statistical significance with the primary results; 8 of 9 models yielded identical conclusions, while one significant finding (Social SS – Mullen EL) became marginal (*p* = 0.142) (data not shown). *post-hoc* pairwise comparisons indicated all group differences were significant for Speech SS – Mullen RL and EL (all *p* < 0.05).

#### Speech composite latent classes

Speech composite class membership was associated with 24-month receptive and expressive language outcomes (*p*s<0.001), including when controlling for maternal education and diagnosis. On average, Class 1 exhibited delays in both expressive and receptive language at 24 months, Class 2 had typical-to-slightly-advanced 24-month expressive and receptive language age equivalent scores, and Class 3 had mean receptive and expressive language age equivalent scores roughly consistent with 24-month age-based norms (Table 6).

#### Symbolic composite latent classes

Symbolic composite class membership was associated with 24-month receptive and expressive language outcomes (*p*s<0.001) after controlling for maternal education and diagnosis, with Class 2 on average exhibiting elevated 24-month RL and EL AE scores, and Class 1 being associated with delays in both RL and EL age equivalents (Table 6).

### Associations between social communication latent classes and school-aged social disability

On average, SRS-2 total T scores in the sample indicated mild to no social impairment concerns across the sample. Social, speech, and symbolic latent class membership was associated with school-age (5–7 year old) social disability such that, on average, children in Class 2 tended to have lower levels of social impairment compared to those in Class 1 (and children in speech composite Class 3 had SRS total T scores intermediate between those in Classes 2 and 1). However, these associations were not robust to controls for maternal education and diagnosis (Table 7).

**Table 7:** Social Responsiveness Scale (SRS-2) outcomes by latent class solutions, unadjusted and adjusted for demographic and clinical class differences (maternal education and child diagnosis).

Outcome	Unadjusted, LS-Mean (95% CI)				Adjusted, LS-Mean (95% CI)			
	Class 1	Class 2	Class 3	*P*-Value	Class 1	Class 2	Class 3	*P*-Value
Social SS	59.7 (51.3, 68.1)	47.1 (45.5, 48.7)	NA	**0.004**	57.3 (48.2, 66.4)	55.0 (50.4, 59.7)	NA	0.649
Speech SS	56.4 (47.2, 65.5)	45.3 (43.5, 47.1)	48.8 (46.4, 51.3)	**0.009**	59.4 (51.8, 66.9)	54.0 (49.1, 58.8)	53.6 (49.3, 57.9)	0.327
Symbolic SS	55.4 (49.2, 61.5)	46.5 (45.1, 48.0)	NA	**0.007**	55.1 (49.0, 61.2)	53.6 (47.2, 60.0)	NA	0.650

^a^ LS-means and 95% CI are based on linear regression models. “Adjusted” models control for maternal education and child diagnosis. SRS outcomes: *N* = 117. NA = class not present for the outcome measure. CSBS assessment covers years 2007–2020. In a sensitivity analysis restricted to participants with complete covariate data, adjusted associations were consistent in strength, direction, and statistical significance with the primary results (data not shown). For SRS, unadjusted significance was driven by LCA Class 1 vs. Class 2, while the adjusted model showed no significant pairwise differences (consistent with the omnibus test *p* = 0.327).

## Discussion

This study identified latent classes of early social communication development (modeled as CSBS social, speech, and symbolic composite standard scores) in a large heterogeneous sample of infants and toddlers who had been characterized and assessed longitudinally between 12 and 24 months of age. LCA and GMM approaches identified two distinct classes of development in the social and symbolic domains—a lower average magnitude, roughly decreasing trajectory (Class 1), and a higher, average magnitude, roughly increasing trajectory (Class 2). Similar classes (1 and 2) were also identified in the speech domain, along with a third, intermediate magnitude, parabolic trajectory (Class 3), which was the largest of the latent classes within this domain. It should be noted that for each domain, entropy values were approaching but did not reach the recommended threshold of 0.80 (social = 0.66; speech = 0.63; symbolic = 0.70; 63), indicating some overlap in class assignment. The resulting classes, however, were clinically meaningful and we consider them to be useful developmental profiles as opposed sharply delineated trajectory groups. Associations between latent class membership and child diagnostic classifications revealed that, across all social communication domains, the lower/decreasing class (Class 1) was comprised predominantly of children with outcome diagnoses of ASD (social = 74.2%, speech = 79.7%, symbolic = 62.3%), while the majority of children in the higher/increasing class (Class 2) were TD (social = 60.8%, speech = 75.5%, symbolic = 63.9%). In the speech domain, the medium/parabolic class (Class 3) was comprised of roughly equal proportions of TD (36.5%) and non-AUT children (34.5% cumulative across all categories), and a lower proportion of children diagnosed with ASD (26.5%).

With regard to sociodemographic characteristics across all domains, the lower/decreasing class tended to be majority male, and have higher proportions of Black and Multi-racial or Other Race children than the higher/increasing class, which had higher proportions of White children and higher maternal education (particularly advanced degrees) compared to the lower/decreasing class. In the speech domain, the medium/parabolic class was predominantly male, and intermediate between classes 1 and 2 in terms of maternal education, and proportions of Black, White, and Multi-racial or Other Race children.

Across all domains, standard scores between the classes differed significantly at all timepoints, from 12 to 24 months of age. Our analytical approach thus allows us to identify differences in social communication skill development earlier than is possible from taking a diagnostic group differences-based approach [as in (11), who detected diagnostic differences in CSBS composite scores by 20 months]. Our focus on social communication development also enabled us to identify significant differences between latent classes much earlier in development than was possible using multilevel growth curve analysis of cognitive and language scores in siblings of children with and without ASD, followed from 4 months to 7 years of age (57). We identified fewer clinically meaningful latent classes than some past work investigating autism symptomatology using similar methods, likely due to our narrower focus on social communication (rather than the spectrum of ASD symptoms, including restricted and repetitive behaviors), and perhaps due to our focus on the 12–24 month period, in contrast to these studies of ASD symptomatology in children from 9 months to 4 years of age, which reveal trajectories that increasingly diverge over time (64, 65). Our findings in the social domain, which comprises emotion and eye gaze, communication, and gestures subscales, and the symbolic domain, which comprises understanding and object use, also complement and align with past research in which infants later diagnosed with ASD were found to exhibit fewer eye gaze, facial expression and gesture skills as young as 9 months, and understanding and object use differences by 12 months (19). In the speech domain, which considers both sounds and words produced, the decreasing class had lower scores than the increasing class, and the medium/parabolic class had scores intermediate between the other two classes across all timepoints from 12 to 24 months [although differences between Classes 1 and 3 drop to only marginally significant (*p* = 0.057) at the 15 month timepoint; see Supplementary Table S3]. These results align with those of Longard et al. (54), who identified three expressive and receptive language trajectory classes between 6 and 36 months of age, although their intermediate classes contained the highest proportions of children with ASD. In the current analysis, Speech domain Class 3 has proportions of ASD and TD children intermediate between classes 1 and 2, but the highest cumulative proportion of non-AUT children of all the classes.

Although these results indicate general trends across all 3 social communication domains examined—wherein higher magnitude increasing skill trajectories starting around 12 months of age (Class 2) tend to be associated with higher social functioning or lower social disability, and lower magnitude decreasing trajectories evident from 12 months of age tend to be associated with higher levels of social and other disabilities (Class 1)—class trajectories differed in shape across social communication domains. For example, Class 2 average trajectories in the social domain appear to increase steadily from about 12 to 18 months, then plateau or even decrease slightly between 18 and 24 months, whereas symbolic Class 2 average trajectories continue to increase (although curvilinearly) through to 24 months; average social Class 1 trajectories appear to slowly and steadily decline from 12 to 24 months, whereas average symbolic Class 1 trajectories are roughly steady from 12 to 16 months then show steeper declines between 16 and 24 months. In the speech domain, where 3 distinct classes were identified, Class 2 trajectories increase and Class 1 trajectories decrease steeply and linearly from 12 to 24 months, so that the average magnitude of these two classes diverges rapidly over this time period; Class 3 trajectories, of intermediate average magnitude across the entire 12 to 24 month range, appear to hold steady or slightly decrease from 12 to 16 months of age, then slowly increase from 16 to 24 months of age. Taken together, these results suggest the importance of differentially assessing and monitoring social, speech, and symbolic component skills for determining the most efficacious targets of early interventions aimed at improving social communication and promoting positive social and/or language development.

With regard to language development, class membership was also significantly associated with language outcomes at 24 months across all domains, even after controlling for diagnosis and maternal education. For the social and symbolic composites, the lower/decreasing class had, on average, delayed receptive and expressive language outcomes. Speech domain class membership was significantly associated with language outcomes at 24 months, controlling for diagnosis and maternal education, such that on average, the higher/increasing class had receptive and expressive language outcomes that roughly aligned with age-based norms, the lower/decreasing class had delayed receptive and expressive language, and the medium/parabolic class had slightly delayed expressive language, but on-target receptive language compared to age-based norms. These results again indicate both general and specific trends across social communication domains—in general, lower levels of social communication skills evident from around the first birthday (and declines thereafter) indicate increased likelihood of later language delays, even after controlling for the specific influence of diagnosis, and/or maternal education (our indicator of socioeconomic status); this relationship appears to be most apparent for the speech domain, wherein membership in the lower/decreasing class is associated with the lowest 24-month receptive and expressive outcomes, and wherein lower/decreasing or medium/parabolic class membership are associated with lower expressive than receptive outcomes at 24 months.

Few studies to date have linked very early social development with long-term (e.g., school age) outcomes. The current study represents a step toward these efforts, although the subsample for which this was possible was a small proportion of our overall sample. Across all domains of social communication profiled herein, class membership was associated with school-aged social disability scores, the higher/increasing class trajectories were associated with lower levels of social disability, lower/decreasing class trajectories predicted higher average social disability, and Speech medium/parabolic class trajectories predicted social disability intermediate to Classes 1 and 2. After controlling for diagnosis and maternal education however, these differences did not remain statistically significant.

Another significant contribution of this study is our inclusion of non-AUT samples, which to date have been examined to a limited extent in comparison to ASD and TD groups. The analyses herein demonstrate that children with known genetic disorders were present in higher proportions in the lower/decreasing class than the higher/increasing class across all social communication domains—a similar pattern observed as that for children with ASD. In contrast, within the social domain, children diagnosed as subthreshold ASD or BAP, DD, or having psychiatric conditions were present in higher proportions in the higher/increasing class—a similar pattern as TD children; in the symbolic domain, subthreshold ASD/BAP children were present in higher proportions in the higher/increasing class (like TD children) and DD children were present in higher proportions in the lower/decreasing class. In the speech domain, DD and subthreshold ASD/BAP children were present in highest proportions in the medium/parabolic class (and children with psychiatric conditions were roughly evenly distributed across the higher/increasing and medium/parabolic classes). At a high level, these patterns may indicate a continuous distribution of social communication skills across the population, where children with ASD, on average, tend to show lower performance on these skills, TD children on average exhibit relatively highest performance on these skills over development, and non-AUT children likely showing varying levels of proficiency across social communication domains, between these two extremes. These results do not appear to suggest profiles of social communication development specific to ASD. However the results also indicate potential composite-specific patterns: children with ASD and known genetic disorders predominantly show trajectories of social development that fall into the lower/decreasing class, while all other groups predominantly follow the higher/increasing trajectory; within the domain of speech, DD and subthreshold ASD/BAP children predominantly fall into a medium/parabolic class, and thus, on average, have distinct speech developmental trajectories from both ASD and TD children; symbolic development appears to better distinguish children with subthreshold ASD/BAP and ASD, but children with ASD and DD may show more similar patterns of development in this domain.

### Limitations and future directions

Although this study takes advantage of a very large sample of infants and toddlers who have completed the CSBS, more than half of our sample contributed only one timepoint to the analyses herein. A consequence of this missing data may be misclassification of individuals with fewer timepoints, as is likely reflected in our “medium” (rather than “high”) class entropy values (discussed above). Additionally, although our sample size allowed us to analyze a non-AUT group in comparison to ASD and TD children, some of the non-AUT subgroups contained very few children: children diagnosed as subthreshold DD (*n* = 8) were included in our analyses but not discussed above due to low sample size; those with psychiatric conditions (*N* = 11) and known genetic disorders (*n* = 15) also have relatively low sample sizes and thus their findings should be interpreted with caution.

Additionally, although the current study found significant associations between latent classes of social, speech, and symbolic development and 24-month receptive and expressive language outcomes, the sample for which these language outcomes were available was less than half of the total sample for whom social communication trajectories were modeled, and differed from this total sample in meaningful ways (including racial composition, maternal education, and diagnostic breakdown; see Supplementary Table S5).

This study also included school-age social disability measures in an attempt to examine associations between early social communication development and longer term outcomes; however, the results herein did not indicate a predictive relationship robust to controls for diagnosis and socioeconomic status, between latent classes of early social, speech, or symbolic development and 5–7 year old social outcomes, as measured by the SRS-2. Given an even more limited amount of data available for these analyses however (14.6% of the sample, differing from the total sample on key metrics including maternal education and diagnostic breakdown, see Supplementary Table S6), future work should still aim to examine the predictive value of early social communication trajectories on later social and language outcomes.

### Summary

The findings of this study extend our understanding of social communication development in the second year of life. Across all domains of social communication, differences in skills present at 12 months of age persisted and/or increased through to 24 months. The results herein indicate that, in general, children who show skills consistent with those in the lower/decreasing social, speech, or symbolic domain classes may benefit from interventions targeting these social communication skills from around the first birthday. Such targeted, early social skill interventions have the additional benefit of promoting positive development of both receptive and expressive language abilities throughout the second year of life (and beyond). Children who show trajectories of speech development consistent with those in the medium/parabolic speech class may also benefit from additional monitoring and/or interventions that target precursors to expressive language development in particular.

Additionally, although our findings align with past research indicating lower and declining trajectories of social communication development in ASD relative to TD children, they nonetheless demonstrate complexity and heterogeneity in development across the breadth of social communication skills (as component classes and trajectories differ in number, shape, magnitude, and association with sociodemographic and diagnostic characteristics across social communication domains), as well as heterogeneity within diagnostic categories, which are distributed across classes to different extents across social, speech, and symbolic domains. Early assessment of social communication skills—and interventions or educational services aimed at supporting positive development in potentially vulnerable domains—may thus prove beneficial for children before diagnosis, and/or regardless of familial or other likelihood for autism or other developmental delays and disorders.

## Data Availability

The datasets presented in this study can be found in online repositories. The names of the repository/repositories and accession number(s) can be found below: NDAR collection(s) 2028 and 2767.

## Footnotes

^1^In most cases, CBE diagnoses were assigned at 24 months. Cases with developmental concerns requiring follow-up/confirmatory assessment were invited to complete characterization protocols at 36 months; these protocols involved at least one experienced clinician not involved in the 24-month diagnostic procedures, but took 24-month assessments into account when determining final CBEs. These diagnostic procedures were considered the gold standard for determining diagnostic group membership, as prior research indicates that experienced clinicians’ judgment of children at 24 months is a better predictor of later diagnosis than cut-off scores on the ADOS; and in our group, 24-month CBE diagnostic stability approaches 100% (66–69). Of the 677 children in the current sample for whom evaluation date/age details were available, 69.5% (n=547) had 24-month CBEs and 16.5% (n=130) received confirmatory CBEs at 36 months.

^2^As is typical of longitudinal study designs including infant and child participants, study visits did not always occur at the exact timepoints indicated. For GMM analyses, age at study visit was binned as follows: 12 months=11, 12, 13 month old visits; 15 months=14, 15, 16 month old visits; 18 months=17, 18, 19, 20 month old visits; 24 months=21-30 month visits.

^3^For ease of interpretation maternal education was dichotomized (less than a college degree vs. college degree or higher) for latent class comparisons. However, original response categories were retained for the multivariable models.
