# Supplementary material for: Latent trajectories of early social communication development are associated with autism diagnosis and language outcomes
Source: Front Child Adolesc Psychiatry. 2026 Apr 9;5:1723743. doi: 10.3389/frcha.2026.1723743 (PMC13102786; doi:10.3389/frcha.2026.1723743)
Supplement: Supplementary file 1 [file Datasheet1.pdf]

Supplementary Material

Supplemental Table 1: Comparison of demographic and clinical characteristics between the Emory and Yale cohorts

**Characteristics**	**Emory, N=661 N (%) or Mean (SD)**	**Yale, N=140 N (%) or Mean (SD**	**P-Value**
Child Sex			
Female	235 (35.5%)	51 (36.4%)	0.852
Male	426 (64.5%)	89 (63.6%)	
Child Race			
White	457 (69.1%)	128 (91.4%)	**<0.001**
Black	98 (14.8%)	2 (1.4%)	
Multi-racial or Other	72 (10.9%)	4 (2.9%)	
Asian	18 (2.7%)	5 (3.6%)	
American Indian or Alaskan Native	2 (0.3%)	0 (0%)	
Unknown or Not Reported	14 (2.1%)	1 (0.7%)	
Child Ethnicity			
Hispanic	61 (9.2%)	15 (10.7%)	0.113
Not Hispanic	581 (87.9%)	125 (89.3%)	
Unknown or Not Reported	19 (2.9%)	0 (0%)	
Maternal Education			
HS/GED or Less	75 (11.3%)	0 (0%)	**<0.001**
Associate's degree/Some College	182 (27.5%)	0 (0%)	
Bachelor's degree	135 (20.4%)	0 (0%)	
Master's degree	139 (21%)	0 (0%)	
PhD/Professional degree	66 (10%)	2 (1.4%)	
Unknown or Not Reported	64 (9.7%)	138 (98.6%)	
Age at Mullen (Months), N=366	24.50 (1.15)	24.33 (0.93)	0.119
Age at SRS (Months), N=117	83.94 (9.86)	NA	NA
Diagnosis			
ASD	225 (34%)	16 (11.4%)	**<0.001**
TD	259 (39.2%)	76 (54.3%)	
DD	86 (13%)	18 (12.9%)	
Subthreshold DD	2 (0.3%)	6 (4.3%)	
Subthreshold ASD/BAP	56 (8.5%)	18 (12.9%)	
Psychiatric Conditions	6 (0.9%)	5 (3.6%)	
Known Genetic Disorders	15 (2.3%)	0 (0%)	
Unknown or Not Reported	12 (1.8%)	1 (0.7%)	
Familial Likelihood			
IL	150 (22.7%)	91 (65%)	**<0.001**
LL	317 (48%)	49 (35%)	
Unknown or Not Reported	194 (29.3%)	0 (0%)	

[1]p-values are based on two-sample t-tests (continuous) or Monte Carlo–estimated Fisher's exact tests (categorical)

Across Yale and Emory study sites, participants did not differ on sex, ethnicity, or age at Mullen evaluation. Participant race significantly differed across sites, most notably with greater proportions of Black and Multi-racial or Other participants at Emory (Atlanta, GA), compared to Yale (New Haven, CT) likely due to the demographics of the metropolitan areas in which the study sites were located. Across both sites however, participants were majority White and Non-Hispanic; the specific requirements of the study (i.e., longitudinal, in-person data collection with visits limited to typical business hours), as well as systematic barriers to research participation, likely limited the sociodemographic diversity of our sample (Heffernan et al., 2023; Nathe et al., 2022). Differences in maternal education displayed above are due to the fact that these measures were not systematically collected from Yale participants. The two sites also differ in the proportion of IL and LL infants enrolled (with a higher proportion of the former and a lower proportion of the latter enrolled at Yale, but the reverse at Emory), as well as diagnostic outcomes (with a greater proportion of ASD diagnoses in the Emory vs. Yale cohort), although this may be explained by the relatively high proportion of Emory participants with Unknown or Not Reported familial likelihood. Since the analytic approach of the current manuscript benefits from increased heterogeneity, data from both sites were combined to increase the robustness of the findings herein.

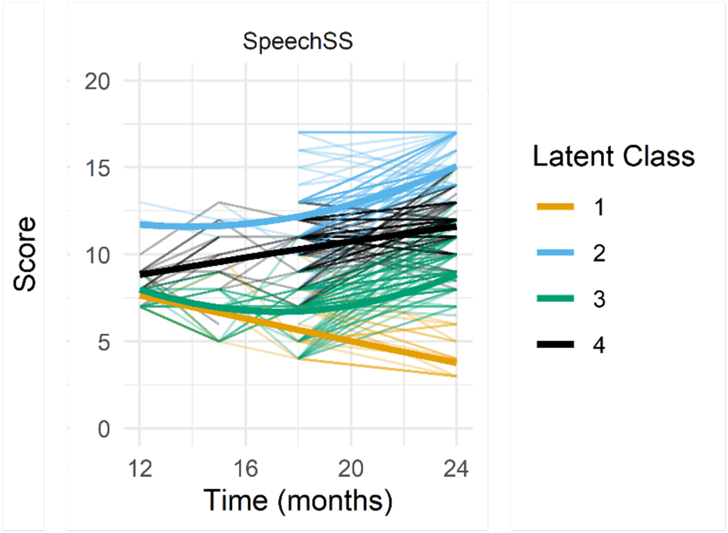

Supplemental Figure 1. Spaghetti plots of 4-class solution for CSBS Speech Standard Score composites with quadratic regression overlay.

Supplemental Table 2: Latent class analysis of 4-class solution for CSBS Speech Standard Score composites, Comparing Classes 2 and 4

**Characteristic**<br>**N (%) or Mean (SD)**	**N**	**Class 2, N=139**	**Class 4, N=199**	**P-Value**
**LCA Features**				
Time 12	151	12.1 (2.0)	9.0 (1.6)	**<0.001**
Time 15	44	12.9 (1.7)	10.2 (1.4)	**<0.001**
Time 18	241	12.2 (1.9)	10.2 (1.4)	**<0.001**
Time 24	239	15.3 (1.3)	11.8 (1.4)	**<0.001**
Class 2 Probability	338	84% (15%)	11% (14%)	**<0.001**
Class 4 Probability	338	15% (15%)	74% (18%)	**<0.001**
Number observations	338	1.8 (0.7)	2.1 (0.8)	**0.004**
**Demographics and Clinical Features**				
Sex	338			0.737
Female		56 (40.3%)	84 (42.1%)	
Male		83 (59.7%)	115 (59.8%)	
Child Race	338			0.093
White		124 (89.2%)	162 (81.1%)	
Black		4 (2.9%)	20 (10.1%)	
Multi-racial or Other		6 (4.3%)	9 (9.5%)	
Asian		3 (2.2%)	7 (3.5%)	
American Indian or Alaskan Native		0 (0%)	0 (0%)	
Unknown or Not Reported		2 (1.4%)	1 (0.5%)	
Child Ethnicity	338			0.604
Hispanic		14 (10.1%)	13 (6.5%)	
Not Hispanic		124 (89.2%)	185 (93.0%)	
Unknown or Not Reported		1 (0.7%)	1 (0.5%)	
Maternal Education	184			0.104
Less than college graduate		10 (20.4%)	45 (33.3%)	
College graduate		39 (79.6%)	90 (66.7%)	
Diagnosis	338			0.600
ASD		8 (5.8%)	11 (5.5%)	
TD		103 (74.1%)	132 (66.3%)	
DD		8 (5.8%)	20 (10.1%)	
Subthreshold DD		2 (1.4%)	4 (2.0%)	
Subthreshold ASD/BAP		15 (10.8%)	21 (10.6%)	
Psychiatric Conditions		2 (1.4%)	5 (2.5%)	
Known Genetic Disorders		0 (0.7%)	3 (1.5%)	
Unknown or Not Reported		1 (0.7%)	3 (1.5%)	
Familial Likelihood	338			0.616
IL		58 (41.7%)	74 (37.2%)	
LL		73 (52.5%)	116 (58.3%)	
Unknown or Not Reported		8 (5.7%)	9 (4.5%)	

Supplemental Table 3: Latent class analysis of Speech SS, post-hoc pairwise comparisons

Characteristic	Post-Hoc Pairwise P-values		
	**Class 1 vs. 2**	**Class 1 vs. 3**	**Class 2 vs. 3**
**LCA Features**			
Time 12	**<0.001**	**0.001**	**<0.001**
Time 15	**<0.001**	0.057	**<0.001**
Time 18	**<0.001**	**<0.001**	**<0.001**
Time 24	**<0.001**	**<0.001**	**<0.001**
Class 1 Probability	**<0.001**	**<0.001**	**<0.001**
Class 2 Probability	**<0.001**	**<0.001**	**<0.001**
Class 3 Probability	0.353	**<0.001**	**<0.001**
Number observations	**<0.001**	**<0.001**	0.242
**Demographics and Clinical Features**			
Sex	**<0.001**	**0.004**	0.094
Child Race	**<0.001**	**0.023**	**<0.001**
Child Ethnicity	**0.048**	0.518	0.259
Maternal Education	**<0.001**	**<0.001**	**<0.001**
Diagnosis	**<0.001**	**<0.001**	**<0.001**
Familial Likelihood	**<0.001**	**<0.001**	**<0.001**

Supplemental Table 4: LCA quadratic regression parameters for each CSBS subscale

	**Social SS**		**Speech SS**		**Symbolic SS**	
**Class**	**Estimate (95% CI)**	**P-Value**	**Estimate (95% CI)**	**P-Value**	**Estimate (95% CI)**	**P-Value**
Social SS						
**Latent Class I**						
N	260		163		313	
Intercept	6.54 (2.96, 10.12)	**<0.001**	12.06 (7.33, 16.78)	**<0.001**	4.41 (0.45, 8.37)	**0.029**
Linear Term	0.05 (-0.39, 0.48)	0.838	-0.40 (-0.99, 0.19)	0.184	0.41 (-0.03, 0.86)	0.069
Quadratic Term	-0.006 (-0.02, 0.01)	0.358	0.003 (-0.01, 0.02)	0.712	-0.018 (-0.03, -0.01)	**0.004**
**Latent Class II**						
N	541		249		488	
Intercept	4.68 (2.28, 7.09)	**<0.001**	10.27 (5.68, 14.85)	**<0.001**	3.81 (1.30, 6.32)	**0.003**
Linear Term	0.51 (0.23, 0.79)	**<0.001**	-0.17 (-0.66, 0.32)	0.494	0.46 (0.16, 0.75)	**0.002**
Quadratic Term	-0.012 (-0.02, -0.01)	**0.002**	0.014 (0.001, 0.03)	**0.040**	-0.007 (-0.02, 0.001)	0.085
**Latent Class III**						
N	NA	--	389		NA	--
Intercept	NA	--	16.63 (12.21, 21.05)	**<0.001**	NA	--
Linear Term	NA	--	-1.11 (-1.66, -0.56)	**<0.001**	NA	--
Quadratic Term	NA	--	0.034 (0.02, 0.05)	**<0.001**	NA	--
Intercept Variance	1.08 (0.58, 1.57)	**<0.001**	0.64 (0.29, 0.99)	**<0.001**	0.83 (0.51, 1.15)	**<0.001**

Supplemental Table 5: Comparison of demographic and clinical characteristics between participants with and without Mullen

Characteristics	No Mullen, N=435 N (%) or Mean (SD)	Mullen, N=366 N (%) or Mean (SD	P-Value
Child Sex			
Female	152 (34.9%)	134 (36.6%)	0.654
Male	283 (64.1%)	232 (63.4%)	
Child Race			
White	273 (62.8%)	312 (85.2%)	**<0.001**
Black	80 (18.4%)	20 (5.5%)	
Multi-racial or Other	56 (12.9%)	20 (5.5%)	
Asian	11 (2.5%)	12 (3.3%)	
American Indian or Alaskan Native	2 (0.5%)	0 (0%)	
Unknown or Not Reported	13 (3%)	2 (0.5%)	
Child Ethnicity			
Hispanic	45 (10.3%)	31 (8.5%)	**0.004**
Not Hispanic	373 (85.8%)	333 (91%)	
Unknown or Not Reported	17 (3.9%)	2 (0.5%)	
Maternal Education			
HS/GED or Less	64 (14.7%)	11 (3%)	**<0.001**
Associate's degree/Some College	116 (26.7%)	66 (18%)	
Bachelor's degree	117 (26.9%)	18 (4.9%)	
Master's degree	69 (15.9%)	70 (19.1%)	
PhD/Professional degree	37 (8.5%)	31 (8.5%)	
Unknown or Not Reported	32 (7.4%)	170 (46.5%)	
Familial Likelihood			
Increased Likelihood	61 (14%)	180 (49.2%)	**<0.001**
Low Likelihood	184 (42.3%)	182 (49.7%)	
Unknown or Not Reported	190 (43.7%)	4 (1.1%)	
Diagnosis			
ASD	185 (42.5%)	56 (15.3%)	**<0.001**
TD	119 (27.4%)	216 (59%)	
DD	77 (17.7%)	27 (7.4%)	
Subthreshold DD	2 (0.5%)	6 (1.6%)	
Subthreshold ASD/BAP	24 (5.5%)	50 (13.7%)	
Psychiatric Conditions	5 (1.2%)	6 (1.6%)	
Known Genetic Disorders	11 (2.5%)	4 (1.1%)	
Unknown or Not Reported	12 (2.8%)	1 (0.3%)	
Site			
Emory	425 (97.7%)	236 (64.5%)	**<0.001**
Yale	10 (2.3%)	130 (35.5%)	

[1]p-values are based on two-sample t-tests (continuous) or Monte Carlo–estimated Fisher's exact tests (categorical)

Supplemental Table 6: Comparison of demographic and clinical characteristics between participants with and without SRS

Characteristics	No SRS, N=684<br>N (%) or Mean (SD)	SRS, N=117<br>N (%) or Mean (SD	P-Value
Child Sex			
Female	243 (35.5%)	43 (36.8%)	0.840
Male	441 (64.5%)	74 (63.2%)	
Child Race			
White	487 (71.2%)	98 (83.8%)	0.082
Black	93 (13.6%)	7 (6%)	
Multi-racial or Other	69 (10.1%)	7 (6%)	
Asian	19 (2.8%)	4 (3.4%)	
American Indian or Alaskan Native	2 (0.3%)	0 (0%)	
Unknown or Not Reported	14 (2%)	1 (0.8%)	
Child Ethnicity			
Hispanic	68 (9.9%)	8 (6.8%)	0.094
Not Hispanic	597 (87.3%)	109 (93.2%)	
Unknown or Not Reported	19 (2.8%)	0 (0%)	
Maternal Education			
HS/GED or Less	73 (10.7%)	2 (1.7%)	**<0.001**
Associate's degree/Some College	150 (21.9%)	32 (27.4%)	
Bachelor's degree	127 (18.6%)	8 (6.8%)	
Master's degree	87 (12.7%)	52 (44.4%)	
PhD/Professional degree	49 (7.2%)	19 (16.2%)	
Unknown or Not Reported	198 (28.9%)	4 (3.4%)	
Familial Likelihood			
Increased Likelihood	211 (30.8%)	30 (25.6%)	**<0.001**
Low Likelihood	281 (41.1%)	85 (72.7%)	
Unknown or Not Reported	192 (28.1%)	2 (1.7%)	
Diagnosis			
ASD	229 (33.5%)	12 (10.3%)	**<0.001**
TD	250 (36.5%)	85 (72.7%)	
DD	96 (14%)	8 (6.8%)	
Subthreshold DD	8 (1.2%)	0 (0%)	
Subthreshold ASD/BAP	67 (9.8%)	7 (6%)	
Psychiatric Conditions	9 (1.3%)	2 (1.7%)	
Known Genetic Disorders	13 (1.9%)	2 (1.7%)	
Unknown or Not Reported	12 (1.8%)	1 (0.8%)	
Site			
Emory	544 (79.5%)	117 (100%)	**<0.001**
Yale	140 (20.5%)	0 (0%)	

[1]p-values are based on two-sample t-tests (continuous) or Monte Carlo–estimated Fisher's exact tests (categorical)
